# Clinical Evaluation of an Integrated Tri‐Modal Transdermal Device for Enhanced Serum Absorption

**DOI:** 10.1111/jocd.70674

**Published:** 2026-01-21

**Authors:** Youjin Kim, Seongwon Hong, Kiyoung Chang, Seoyeon Han, Huijun Leung, Jee Sun Lee, Soha Jeon, Hae Jo

**Affiliations:** ^1^ Research and Development Amway Corporation Seoul Republic of Korea

Physical transdermal delivery systems such as electroporation, sonophoresis, and iontophoresis have been studied for their potential to enhance cutaneous absorption of active ingredients, improving penetration and bioavailability in cosmetic and therapeutic applications [1]. Electroporation delivers short high‐voltage pulses that transiently disrupt the stratum corneum, forming micropores that allow passage of otherwise impermeable molecules [2, 3]. Sonophoresis employs ultrasound waves (~1 MHz) to disorder intercellular lipid bilayers, widening diffusion pathways [4]. Iontophoresis applies a low‐level electrical current to drive charged actives via electro‐repulsion and promote nonionic transport through electroosmosis [5].

Combining physical enhancers is suggested to lead to additive or potentially synergistic improvements in transdermal flux [6]. Previous studies have shown that combining two modalities enhances penetration more than either alone—for example, sonophoresis can lower the electroporation threshold and increase skin permeability, allowing iontophoresis to subsequently provide a sustained electrical driving force that maintains or augments flux [1, 3, 6]. Although such dual‐modality combinations have been explored, no clinical study has evaluated the integrated use of all three modalities or quantified tri‐modal delivery using depth‐resolved confocal Raman spectroscopy, particularly in consumer‐grade devices.

This study used depth‐resolved confocal Raman spectroscopy to assess penetration depth and concentration of a tri‐modal delivery system compared with manual application. In this open‐label crossover study (IRB approval: Ellead No. 240912T005), 22 healthy Korean women aged 23–60 years (mean ± standard deviation: 47.6 ± 9.4) without history of skin diseases were enrolled. Following a 30‐min acclimatization (20°C–22°C; 40%–60% humidity), each participant received a 90‐s serum application on one forearm. On the contralateral forearm, the same serum was applied followed by a single device application (electroporation + sonophoresis + iontophoresis). Device parameters were 9.2 V and 1 kHz for electroporation, 5 V and 1 kHz for iontophoresis, and 1 MHz ultrasound for sonophoresis, selected based on preclinical optimization and established safety‐validated ranges in cosmetic skin‐delivery applications. The serum contained plant‐based collagen, cranberry biopeptides, fermented oligopeptides, and white chia peptides in a liposomal carrier. Raman spectroscopy (gen2 Skin Composition Analyzer; River Diagnostics, Rotterdam, Netherlands) was performed at baseline and 30 min after application, with 2 μm steps to 30 μm. Penetration quantity was expressed as Raman intensity relative to keratin (A.U./cm^2^), and depth (μm) defined as the deepest point exceeding baseline. Statistical analyses were performed in SPSS version 27 (IBM, Armonk, NY, USA). Within‐subject (pre‐ vs. post‐application) comparisons were analyzed using the paired *t‐*test, and intraindividual contralateral comparisons (serum‐only vs. device‐assisted serum application) were evaluated using the Wilcoxon signed‐rank test, with significance set at *p* < 0.05.

Both conditions demonstrated significant within‐subject increases in penetration metrics (device‐assisted serum application, *p* < 0.001; serum‐only, *p* < 0.001; data not shown). Contralateral comparison confirmed that device‐assisted serum application resulted in significantly greater delivery, with mean absorption increasing from 0.080 ± 0.019 A.U./cm^2^ at serum‐only sites to 0.207 ± 0.145 A.U./cm^2^ at device‐assisted sites (*p* < 0.001). Mean penetration depth increased from 7.36 ± 1.99 μm to 17.00 ± 5.16 μm (*p* < 0.001; Figure 1). Raman maps (Figure 2) corroborated these findings: serum‐only application showed narrow signals within 6–10 μm of the stratum corneum, whereas device‐assisted application exhibited broader bands extending to 12–26 μm, reaching the upper viable epidermis. Findings were consistent across participants, and safety monitoring—via dermatologist‐assessed visual inspection (e.g., erythema, itching, stinging) and participant‐reported sensations—revealed no adverse reactions or discomfort.

**FIGURE 1:**
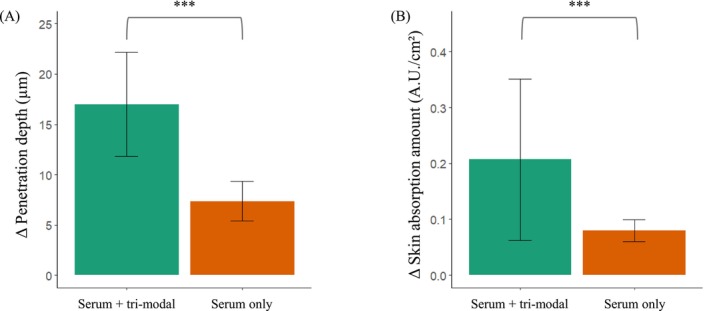
Device‐assisted tri‐modal application (electroporation + sonophoresis + iontophoresis) significantly increases both the penetration depth and quantity of serum delivery (*n* = 22). Change (Δ) in (A) penetration depth (μm) and (B) amount of skin absorption (A.U./cm^2^). Bars represent means ± standard deviations; ***p < 0.001, Wilcoxon signed‐rank test was used for intraindividual (contralateral arm) comparison between serum‐only and serum + tri‐modal device application.

**FIGURE 2:**
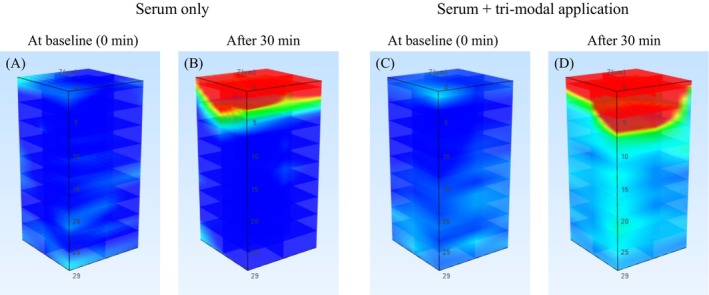
Depth‐resolved Raman maps of serum penetration in paired forearms of a representative subject. (A, B) Control forearm applied serum only at baseline (0 min) and 30 min after application, respectively. (C, D) Test arm applied serum plus a single device‐assisted tri‐modal application (electroporation + sonophoresis + iontophoresis) at the same time points. Maps were acquired with dual‐wavelength excitation—785 nm (fingerprint, 400–2500 cm^−1^) and 671 nm (high‐wavenumber, 2500–4000 cm^−1^). The color scale (shared across panels) represents relative Raman intensity; red indicates higher serum concentration.

Direct cross‐study comparison of device‐assisted transdermal delivery systems is inherently limited, as enhancement outcomes are highly influenced by differences in device settings, application protocols, anatomical sites, study designs, and endpoint measurement methodologies [7]. Therefore, we applied a within‐study comparison to the serum‐only control, in which the tri‐modal device demonstrated a 2.6‐fold increase in penetration quantity and a 2.3‐fold increase in depth. While a clinical study using a dual‐modality approach (electroporation + sonophoresis) demonstrated an approximately 2–3‐fold improvement versus passive application [8], the enhancement observed in the present study is considered clinically meaningful. This level of enhancement was achieved after a single 90‐s session without adverse effects, suggesting improved access to metabolically active keratinocytes and the potential for greater cosmetic benefit through efficient intradermal delivery. Electroporation, sonophoresis, and iontophoresis—acting via transient pore formation, lipid bilayer perturbation, and electrophoretic transport—appear complementary and potentially synergistic in promoting deeper absorption of active ingredients, although confirmatory studies using single‐ and dual‐modality comparators are warranted. This clinical finding provides quantitative evidence supporting the safety and efficacy of low‐energy tri‐modal delivery technology and underscores its potential for enhancing at‐home cosmetic performance.

## Author Contributions

S.J. and H.J. conceptualized the research. S.H. performed project administration. K.C., S.H., H.L. and J.S.L. contributed essential materials or tools. S.H. and Y.K. interpreted the data. Y.K. wrote the paper.

## Ethics Statement

The study was reviewed and approved by the Ethics Committee of Ellead (IRB No. 240912T005) on October 04, 2024. This study was conducted according to the guidelines of the Korea Ministry of Food and Drug Safety on clinical and efficacy testing of cosmetics and the Standard Operating Procedures (EL‐P‐7400) of Ellead. All patients signed informed consent on the procedure.

## Conflicts of Interest

All authors are full‐time employees of Access Business Group North Asia. The study was funded by the employer. Apart from their regular salaries, the authors received no additional compensation, stock, or royalties related to this work. The sponsor had no role in study design; data collection, analysis, and interpretation; manuscript preparation; or the decision to submit. The authors retained full control over manuscript preparation and submission.

## Data Availability

Research data are not shared.
